# An elderly diabetic patient with McArdle disease and recurrent rhabdomyolysis: a potential association with late hypoinsulinemia?

**DOI:** 10.1186/s12877-020-01812-4

**Published:** 2020-11-05

**Authors:** Pedro Renato Chocair, Sara Mohrbacher, Precil Diego Miranda de Menezes Neves, Leonardo Victor Barbosa Pereira, Erico Souza Oliveira, Luciana Loureiro Nardotto, Alessandra Martins Bales, Victor Augusto Hamamoto Sato, Sabrina Neves Silva, Bernadete Maria Coelho Ferreira, Américo Lourenço Cuvello-Neto

**Affiliations:** 1 Internal Medicine Service, Oswaldo Cruz German Hospital, Rua Treze de Maio, 1815 – Bairro Bela Vista, São Paulo, SP 01323-020 Brazil; 2 Nephrology and Dialysis Center, Oswaldo Cruz German Hospital, São Paulo, Brazil; 3 Nephrology Division, University of São Paulo School of Medicine, São Paulo, Brazil

**Keywords:** Rhabdomyolysis, Acute kidney injury, McArdle disease, Glycogen storage disease type V, Diabetes, Hypoinsulinemia

## Abstract

**Background:**

McArdle disease is a myopathy caused by mutations in *PYGM* gene that is characterized by reduced or absent activity of myophosphorylase. Reports of patients with concomitant McArdle disease and diabetes are scarce. We report a case of a patient with a late diagnosis of McArdle disease and we postulate that symptoms may be related to hypoinsulinemia.

**Case presentation:**

This report describes the evolution of an elderly diabetic patient with confirmed diagnosis of McArdle’s disease based on the absence of myophosphorylase activity in the analysis of muscle biopsy, and a homozygous mutation in the *PYGM* gene. The variant – Chr11: 64.525 (p. Asn168*fs) has not been previously described. The diagnosis of McArdle disease was confirmed after two episodes of rhabdomyolysis, at 77 and 81 years of age, as the symptoms were, until then, discrete. The “second-wind phenomenon” was not spontaneously reported, but it was confirmed when directly questioned. We postulate that the later episodes of rhabdomyolysis occurred because of a progressive decrease in insulin production with a consequent reduction in the uptake of blood glucose by muscle cells, thus compromising the cellular energy balance. To our knowledge, this is the first report of recurrent rhabdomyolysis in an elderly diabetic patient with genetically proven McArdle disease. Our initial attempt to reduce insulin resistance with metformin and pioglitazone was not effective, possibly because of inadequate insulinemia. However, an improvement was evident after the administration of low doses of intermediate-acting insulin.

**Conclusions:**

In view of the patient’s clinical evolution, we suggest the use of medication that reduces insulin resistance for patients with McArdle disease and type 2 diabetes, pre-diabetes or even normoglycemic metabolic syndrome.

## Background

McArdle disease is one of the best known, genetically-determined myopathies. The condition was described originally by Brian McArdle in 1951, for a patient with exercise-induced myalgia who failed to show increased blood lactate levels during physical exercise [1]. The disease was later associated with myophosphorylase deficiency [2–4] caused by mutation *PYGM* gene [5]. McArdle disease is also known as glycogen storage disease (GSD) type V, to differentiate it from a number of other myopathies such as GSD-II (Pompe disease), VII, VIII, IX, X, XI, and XIII, each presenting a specific enzyme deficiency tied to different stages of glycogenolysis [6], in addition to mitochondrial myopathies that manifest primarily with chronic progressive external ophthalmoplegia and ptosis [7].

This case report discusses the clinical progression of a diabetic patient diagnosed with McArdle myopathy at the age of 81, by which time he had experienced a second episode of rhabdomyolysis exactly 4 years after his first episode. The patient had suffered mild symptoms since his youth, including the “second wind phenomenon,” confirmed when he was specifically asked about it.

## Case presentation

An 81-year-old male patient presented at our service complaining of marked adynamia, and reported that his urine had been darker than normal for the past 2 weeks. Symptoms had started with moderate, self-limiting diarrhea lasting for 3 days, without fever or signs of disease in his stools, and moved to progressive muscle weakness. Ten days before seeking medical help, the patient had fallen and bruised his scalp. Clinical examination and workup did not find any repercussions from the incident. The patient had no respiratory, urinary, or digestive complaints. According to his wife, the patient had suffered bouts of muscle weakness after having short walks since his youth. He has never been on statins. When asked about it, the patient reported his muscle symptoms improved after a few minutes of rest.

He had been previously diagnosed with late-onsetdiabetes mellitus (prescribed oral hypoglycemic drugs), mild to moderate cognitive impairment of unknown origin, benign prostatic hyperplasia, and had undergone a cholecystectomy. He was on the following medications: tamsulosin 0.4 mg/day, vitamin D3 1000u/day, ASA 100 mg/day, vitamin B12 5000 IU/week, gliclazide 30 mg/day, memantine 10 mg/day, rivastigmine 27 mg/day, linagliptin 5 mg/day, and venlafaxine 75 mg/day. His family had a history of consanguineous marriages.

The patient was well-oriented during physical examination. His blood pressure was normal (130/80 mmHg) and he was afebrile. He did not present additional noteworthy clinical findings.

The patient had been hospitalized 4 years previously (2015), for myalgia with nausea, vomiting, and decreased urine output. He had been exposed to elevated temperatures during a fishing trip to the Amazon region. He was transferred to our Center and arrived with pronounced acute renal failure, signs of uremia, and findings consistent with rhabdomyolysis (Table 1). His workup showed a serum myoglobin level of 5351 ng/mL (normal range: < 72 ng/mL); the patient was positive for urine myoglobin and negative for anti-Leptospira and anti-Plasmodium antibodies. He was promptly prescribed renal dialysis therapy, undergoing three hemodialysis sessions, and recovered fully from renal impairment. The patient was not tested for viral infection and the cause of severe dehydration was attributed to a potential case of rhabdomyolysis.

**Table 1:** Laboratory findings in the patient’s two hospital admissions

Laboratory tests	Normal range	2015 - First hospitalization								2019 - Second hospitalization				
		11/22	11/22	11/23	11/25	11/26	11/27	11/28	12/01	07/28	07/29	07/30	08/01	08/03
Creatinine	0.7–1.3 mg/dL	9.9	10.1	–	5.2	6.1	6.0	5.5	2.9	1.03	–	0.81	0.84	–
Urea	10–50 mg/dL	232	221	–	86	68	–	91	77	48	–	–	33	–
AST	≤ 40 U/L	281	–	–	–	–	35	–	–	70	–	63	26	–
ALT	≤ 41 U/L	171	–	–	–	–	47	–	–	68	–	71	42	–
CK	38–174 U/L	22,800	15,545	7067	2516	1742	1294	866	384	5141	4373	2716	468	228

**Fig. 1:**
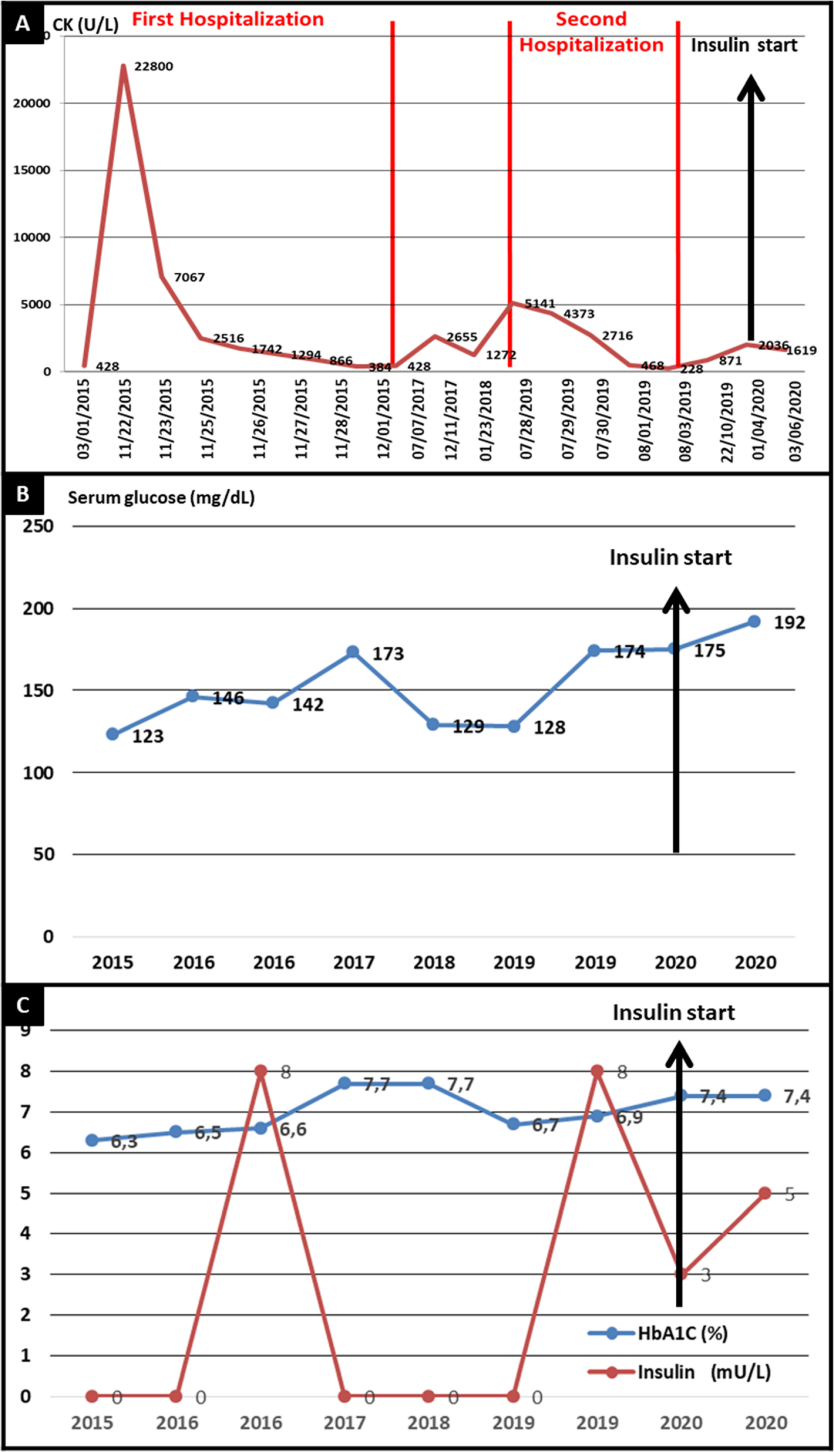
Patient laboratory tests along followup. **a** Creatine kinase (CK) levels following his first hospitalization. **b** Serum glucose levels and **c** HbA1c and Insuline levels. Black arrows indicate the time point of insulin start

The patient was asymptomatic between 2015 and 2019 and performed supervised physical activities with the aid of a personal trainer. Figure 1a shows that his creatine kinase (CK) levels had been consistently above normal range, and may have remained unremarked on account of his good overall clinical condition.

His workup at the time of hospitalization (Table 1) showed elevated blood glucose and glycated hemoglobin levels, and normal renal function. The patient, however, had another episode of rhabdomyolysis, without apparent cause, as evidenced by high serum CK and plasma myoglobin (364 ng/mL) levels. The patient was examined for viral infections, by Polymerase chain reaction (PCR) testing for Epstein-Barr virus, antibodies against Anti-Coxsackie A and B viruses, and detection of enterovirus RNA in peripheral blood. Results were all inconclusive.

Symptoms of muscle fatigue since a young age, in combination with the “second wind phenomenon,” recurrent rhabdomyolysis without an apparent cause, persistent elevation in CK levels, a family history of consanguineous marriage, and negative test results for viral infection built a strong case around a potential hereditary myopathy. As a part of additional investigation, the patient underwent a biopsy of the brachial biceps. With regard to morphology, no change was seen on the size of muscle fibers or in connective tissue and endomysial/perimysial fat. No signs of centronuclear myopathy, fiber necrosis, macrophages, or lipid accumulation were found. Subsarcolemmal vacuoles were observed, along with some with PAS-positive material. Myophosphorylase histochemical staining was negative.

Supplementary diagnostic workup included genetic analysis via next-generation sequencing with a gene panel for muscular dystrophies, myopathies, and myasthenia gravis, which revealed a homozygous insertion at Chr11:64.525.744 T > TA in the gene *PYMG* causing a frameshift mutation and the introduction of a stop codon halting translation (p.Asn168*fs). This mutation has not been described in medical literature.

The combination of clinical, histology, and genetic-molecular findings confirmed the diagnosis of McArdle disease. The patient was discharged on a prescription of metformin 1000 mg/day and pioglitazone 30 mg/day. His workup had not improved 2 months after the start of therapy (Fig. 1b and c), at which time his prescription was changed to low dose intermediate-acting insulin. The patient and his family have since reported significant clinical improvement.

## Discussion and conclusions

Myophosphorylase is the first enzyme involved in glycogenolysis [6]. Therefore, the absence of myophosphorylase disrupts glycogen utilization and compromises the production of energy in muscle cells, thereby producing muscular symptoms such as rhabdomyolysis [8].

Carbohydrate intake before engaging in physical exercise helps to improve muscle performance of individuals with McArdle disease by improving the supply of glucose – and energy – to muscle fibers, thus compensating for the energy deficit associated with reduced glycogenolysis [5, 6, 9–13]. Since there is no involvement of liver or heart glycogen phosphorylase, the symptoms are limited to striated muscle activity [6, 12].

The clinical symptoms manifested in patients with McArdle include muscle fatigue and cramps, particularly at the start of physical activity. Nearly all patients suffer from the “second wind phenomenon,” a finding described by Pearson [7] deemed a pathognomonic sign of McArdle disease [2], in which muscle performance improves after a few minutes of rest into physical activity [10, 13]. Myoglobinuria occurs in about half of the patients after more intense physical exercise, while many of these patients then developing acute kidney failure [12, 14]. Persistent CK level elevation even during extended inactivity is a characteristic finding in individuals with McArdle [6]. Symptoms usually start between the second and third decades of life, although earlier and later onset cases have been reported in association with differences in living and eating habits [9]. It is unclear why functional capacity deteriorates more sharply in women than in men with the disease [6].

The estimated prevalence of GSD-V varies significantly between regions and studies, with reports ranging from 1:100000 to 1:170000 [6, 9]. Discrepancies have been ascribed to diagnostic barriers, since in addition to clinical acumen, McArdle disease requires relatively complex tests available in a limited number of centers. Additionally, mild clinical manifestations may often be underappreciated.

The combination of McArdle and diabetes has been sparsely cited in medical literature. In 1966, Yamauchi *et al.* [10] reported the case of a 64-year-old, obese, type 2 diabetic patient with McArdle, who did not present with the classical signs of the disease. This was attributed to increased utilization by muscle cells of the elevated blood glucose. Thus insulin resistance can offset the symptoms usually seen in McArdle individuals. In other words, high insulin and glucose levels may mask the typical progression of patients with McArdle disease, as seen in the case reported by Yamauchi and coworkers. Mineo *et al*. [15] reported that increased blood glucose and insulin levels following infusion may further induce glycolysis in muscle tissue, a fact to be considered in patients with significant insulin resistance. Ross and Goldner [16] described the importance of providing parenteral glucose supplementation to a morbidly obese patient submitted to bariatric surgery, to counteract the decrease in carbohydrate intake caused by the procedure. Proper energy supply to muscle cells to prevent rhabdomyolysis was maintained and reinforced with intravenous glucose during and after surgery. We propose that high insulinemia (commonly seen in patients with type 2 diabetes caused by the high insulin resistance) and hyperglycemia were beneficial to this patient by promoting an adequate supply of blood glucose to the muscle fiber. Currently, lifestyle changes that include a high carbohydrate diet and regular physical activity are recommended for McArdle patients. However, for elderly and diabetic patients, as in the case presented, these measures need to be individually analyzed, as well noted by Ferreira *et al* [17]. Due to the constantly high glycemic level, the addition of carbohydrates becomes a problem as it may cause severe hyperglycemia and its consequences. In this setting, this measure, therefore, should not be recommended for these patients, just as a strict glycemic control. On the other hand, physical activity is limited by the patient's age and requires specialized guidance. We opted to maintain only regular glycemic control, both to prevent hypoglycemia with consequent muscle or systemic complications due to hyperglycemia. Nutritional and pharmacological measures to reduce insulin resistance should be especially valued during the clinical evolution of a patient with metabolic syndrome with or without diabetes.

We were unable to find reports of rhabdomyolysis caused by hypoinsulinemia in patients with diabetes and McArdle disease. The late nature of the two episodes of rhabdomyolysis observed in our patient can be explained by the long-term decrease of insulin levels seen throughout the course of the patient’s type 2 diabetes, which eventually became insufficient to control blood glucose, as evidenced by high levels of glycated hemoglobin. However, hyperglycemia was not able to meet the energy requirements of muscle tissue during two occasions resulting in episodes of rhabdomyolysis.

Thus, probably, the relatively uneventful development of the condition throughout the patient’s life shifted when muscle cell support from blood glucose decreased as a result of insulin deficiency, despite the presence of hyperglycemia. The oral medication prescribed to decrease insulin resistance was ineffective and unable to control the blood glucose. On the other hand, muscle symptoms were alleviated with the introduction of intermediate-acting insulin, thus confirming our hypothesis. The presence of mild, self-limited gastrointestinal symptoms preceding the two episodes of rhabdomyolysis drove our suspicions toward viral infection, but test results were inconclusive.

To summarize, this case is, to our knowledge, the first of its kind in medical literature to describe the unique mutation in the gene *PYGM*, producing bouts of rhabdomyolysis exacerbated by hypoinsulinemia in a patient with longstanding diabetes and mild symptoms. Patients with McArdle disease and diabetes, along with prediabetic individuals with the condition, with adequate blood glucose levels and significant insulin resistance, require additional medical attention. Patients in this group will likely benefit from careful dieting, lifestyle changes. We believe that the reduction of insulin resistance in those patients with McArdle disease accompanied by type 2 diabetes, pre-diabetes or even normoglycemic metabolic syndrome may bring clinical benefits, not observed in our patient due to the late start of insulin-sensitizing medication. In summary, since patients with McArdle’s disease has not limited life expectancy, it is important to keep the diagnosis in mind in symptomatic patients with recurrent CK elevation or rhabdomyolysis.

## Data Availability

All meaningful data generated or analyzed in this study are included in the manuscript.

## Abbreviations

CK: Creatine kinase
GSD: Glycogen storage disease
